# Quality control in resting-state fMRI: the benefits of visual inspection

**DOI:** 10.3389/fnins.2023.1076824

**Published:** 2023-05-04

**Authors:** Rebecca J. Lepping, Hung-Wen Yeh, Brent C. McPherson, Morgan G. Brucks, Mohammad Sabati, Rainer T. Karcher, William M. Brooks, Joshua D. Habiger, Vlad B. Papa, Laura E. Martin

**Affiliations:** ^1^Department of Neurology, University of Kansas Medical Center, Kansas City, KS, United States; ^2^Hoglund Biomedical Imaging Center, University of Kansas Medical Center, Kansas City, KS, United States; ^3^Division of Health Services and Outcomes Research, Department of Pediatrics, Children’s Mercy Research Institute, Kansas City, MO, United States; ^4^Department of Pediatrics, School of Medicine, University of Missouri-Kansas City, Kansas City, MO, United States; ^5^Department of Neurology and Neurosurgery, McGill University, Montreal, QC, Canada; ^6^Department of Population Health, University of Kansas Medical Center, Kansas City, KS, United States; ^7^Bioengineering Program, School of Engineering, University of Kansas, Lawrence, KS, United States; ^8^Department of Statistics, Oklahoma State University, Stillwater, OK, United States

**Keywords:** artifacts, functional magnetic resonance imaging (fMRI), resting state—fMRI, reproducibility of results, quality control

## Abstract

**Background:**

A variety of quality control (QC) approaches are employed in resting-state functional magnetic resonance imaging (rs-fMRI) to determine data quality and ultimately inclusion or exclusion of a fMRI data set in group analysis. Reliability of rs-fMRI data can be improved by censoring or “scrubbing” volumes affected by motion. While censoring preserves the integrity of participant-level data, including excessively censored data sets in group analyses may add noise. Quantitative motion-related metrics are frequently reported in the literature; however, qualitative visual inspection can sometimes catch errors or other issues that may be missed by quantitative metrics alone. In this paper, we describe our methods for performing QC of rs-fMRI data using software-generated quantitative and qualitative output and trained visual inspection.

**Results:**

The data provided for this QC paper had relatively low motion-censoring, thus quantitative QC resulted in no exclusions. Qualitative checks of the data resulted in limited exclusions due to potential incidental findings and failed pre-processing scripts.

**Conclusion:**

Visual inspection in addition to the review of quantitative QC metrics is an important component to ensure high quality and accuracy in rs-fMRI data analysis.

## Introduction

Quality control (QC) in functional magnetic resonance imaging (fMRI) data is a critical step in ensuring accurate interpretation of results and reliable and replicable findings. Data may be corrupted at acquisition due to hardware or software malfunctions, artifacts from metallic objects, spurious physiological signals (heart rate, respiration, etc.) or participant motion. Further, incidental findings of atypical anatomic formations, lesions, or other injury may be grounds for data exclusion if those findings are related to inclusion and exclusion criteria for the study, or if they cause errors in certain processing steps. There is a clear need for consensus on QC approaches for fMRI data, and for a revisiting of reporting standards to improve cross-study interpretation and replicability (Esteban et al., 2017). There are emerging approaches to crowd-source the QC of imaging data sets using a combination of expert curation and a gamified interface for identifying scans. In this paper, we describe our fMRI QC methods from data acquisition through individual preprocessing. Our methods rely on standard tools available through the analysis software we use and also include visual inspection by trained reviewers at multiple stages of the process. While the field recognizes the value of quantitative metrics and automated processes for evaluating data quality, we believe there is added value in qualitative assessment that cannot be captured by quantitative measures of displacement, censoring, or signal intensity or homogeneity. We apply these QC strategies to a publicly available data set and report out standardized outcomes identified in the Frontiers Research Topic, Demonstrating Quality Control (QC) Procedures in fMRI.

Across MRI imaging protocols, fMRI data are particularly sensitive to participant head motion. Strategies exist to minimize participant head motion at data acquisition, such as the use of foam padding around the head, a strap across the forehead, bite bars, or real-time feedback to the participant and prospective motion corrections (Thulborn, 1999; Lazar, 2008; Vanderwal et al., 2015). However, these often require specialized settings, sequences, or equipment and are not sufficient to eliminate all movement and some data will be lost to motion corruption.

One of the most observable effects of head motion on fMRI data is the increase or decrease in signal in the affected volumes. In the case of blood oxygen level dependent (BOLD) imaging, data are acquired in slices through the volume of the brain over the course of a few seconds. The slice to be imaged is excited with a radio-frequency (RF) pulse, and the echo is read out a few milliseconds later. If the excited slice has moved in space, the echo will not be accurately read, leading to reduced signal in that slice. Additionally, the next slice to be acquired may have been excited by the preceding pulse and may have residual signal. A second RF pulse in that slice would lead to increases in signal readout. For these reasons, the volumes surrounding a motion spike are often also unreliable, and these effects may last for several seconds (Power et al., 2014). Compounding this issue is that all voxels within a slice or volume are not likely to be impacted the same way, as motion is rarely limited to translation along a single axis. Because of this, the relationship between signal within a given voxel and motion parameters is not linear (Power et al., 2015). Motion can decrease the fMRI signal temporal stability by causing signal alterations across volumes which eventually increase false outcomes (Satterthwaite et al., 2013). Moreover, motion can potentially modulate connectivity-related measurements because it produces global signal changes resulting in spurious results (Rogers et al., 2007).

Certain populations may be especially prone to movement during fMRI scanning. Children, older people, people with back pain, or people with high impulsivity may not be able to hold still for an entire functional scan, which can last for several minutes (Fox and Greicius, 2010; Couvy-Duchesne et al., 2014; Kong et al., 2014; Couvy-Duchesne et al., 2016; Pardoe et al., 2016). Therefore, the development of new approaches and the optimization of current strategies to reduce motion-related artifacts in fMRI data sets are critical for imaging studies of these populations. Because resting state correlation relies on low frequency modulation within the signal, longer scans are recommended (up to ~10 min in some cases) (Birn et al., 2013), potentially exacerbating the problem of participant movement. Participants may tolerate several shorter scans with breaks in between – collecting multiple resting state scans and concatenating across them improves the signal-to-noise ratio (Chen et al., 2010); however, no strategy completely eliminates the impacts of participant head motion (Power et al., 2014, 2015).

The statistical approach of including motion parameters as nuisance regressors in the analysis reduces the impact of motion and has been widely adopted as a standard processing step (Johnstone et al., 2006). It has been shown that removing, or censoring, only the volumes most affected by motion prior to statistical analysis improves reliability (Power et al., 2012; Carp, 2013; Power et al., 2013). Additional ‘scrubbing’ or removing physiological noise signals is also helpful for removing spurious correlations due to head motion (Siegel et al., 2014). However, censoring alone still has problems. One is how to choose the optimal censoring threshold, which may depend on the level of motion in a data set (Power et al., 2014). Once a threshold has been chosen, another concern is that correlation estimates from participants with reduced data sets after censoring may be noisy or have extreme values that may influence group statistics or reduce power. To address this, many studies also exclude entire participants or scans that exceed pre-specified censoring limits (Power et al., 2015). Excluding participants with greater than 10% censored is often used as a threshold, and less conservative censoring thresholds of 15–25% have been used with pediatric populations (Siegel et al., 2014). An entirely different approach from censoring is to use independent components analysis (ICA) to identify the signal associated with head motion (Griffanti et al., 2014; Siegel et al., 2014; Patriat et al., 2015, 2016; Pruim et al., 2015). Since reliability is dependent on the length of usable data, some researchers exclude participants with usable resting state scan data less than ~5 min after censoring (Van Dijk et al., 2012; Andellini et al., 2015).

Censoring or scrubbing solutions allow for removing motion corrupted data will preserving some data and avoiding excluding entire participant data sets. If motion corruption causes data to not be missing at random, excluding more data in one group than another can cause bias in estimation and result in loss in power or invalid testing procedures (Little and Rubin, 2002). Moreover, excluding acquired data introduces a waste of resources and excessive costs for research services and personnel time. Given the challenge of recruiting well-characterized participants from clinical populations, the commitment of participants, and the cost of data collection and analytic staff, there are financial and social burdens to unnecessarily excluding data.

While motion artifacts have been well-documented to lead to both type I and type II errors in downstream analyses, other issues can and do arise during functional data acquisition and analysis. These include incidental findings of anatomic variability in the images which could indicate a medical concern or a benign anatomic difference that is of little medical concern. These findings, however, could be reason for participant exclusion, for example there is an incidental finding that indicates a previous stroke and stroke is an exclusion criterion for the study. Also, these anatomic variabilities could lead to issues with misalignment or normalization into template space, therefore, visual inspection of the results is warranted. In addition to anatomic variabilities and incidental findings, script failures are another source of error in rs-fMRI data analysis. Analysis of rs-fMRI data is performed as a series of steps, with each step taking the output from the previous step, performing another process, and then generating a new output image. Errors are possible at each step, and it is critical to determine that scripts are performing correctly so that the input–output–input chain does not result in errors in the final output data set. These errors are sometimes difficult to find if one only examines quantitative QC metrics, but can be easy to assess visually, for example if the entire functional series of images have been flipped upside down during processing but are centered with the anatomic image, global metrics of homogeneity will not differ between a correctly aligned and incorrectly aligned image. If such processing errors are allowed into group analysis, the spatial location of anatomy will not match across all participants.

In this paper, we describe our processes for rs-fMRI QC, including review of quantitative and qualitative software-generated metrics and visual inspection at each processing step to ensure the most accurate data are carried forward in the analysis process. Further, we advocate for including as much data as possible to minimize bias and honor the participant time and research resources provided.

## Materials and methods

We performed an analysis of previously published and publicly available human participants’ data provided as part of the Demonstrating QC Procedures in fMRI Research Topic (Biswal et al., 2010; Di Martino et al., 2014; Markiewicz et al., 2021). Briefly, resting state fMRI (rs-fMRI) data were pulled from publicly available datasets (ABIDE, ABIDE-II, functional Connectome Project, Open Neuro) across seven imaging sites, with approximately 20 participants from each site. Imaging parameters are summarized in Table 1. For this Research Topic, the Project leaders renamed the data with new participant IDs and organized them into BIDS common directory format. Each participant had one anatomical image and one or two rs-fMRI sequences. Imaging parameters for the rs-fMRI sequences are reported in Table 1. No information on participant demographics or other characteristics was provided. For the remainder of the paper, we will refer to this data set as the “QC data set.” All procedures involving human participants were performed in accordance with the ethical standards of the Declaration of Helsinki, and the study was approved by the Institutional Review Board where the data were collected. Informed consent was obtained from all participants.

**Table 1:** Resting state fMRI imaging parameters from the seven imaging sites.

Site	Scanner	Field strength	Orientation	In-plane resolution	Spacing between Slices	Repetition time (TR)	Echo time (TE)	Number of Slices	Number of volumes	Parallel reduction (Yes/No)
1	Phillips Achieva	3T	Axial	2.67 mm × 2.67 mm	3.0 mm	2,500 ms	30 ms	47	156	Yes
2	Phillips Achieva	3T	Axial	3.0 mm × 3.0 mm	3.84 mm	2,000 ms	28 ms	38	150	Yes
3	Phillips Achieva DS	3T	Axial	2.56 mm × 2.56 mm	3.1 mm	2,500 ms	30 ms	45	162	Yes
4	Unknown	3T	Unknown	2.67 mm × 2.67 mm	3.0 mm	2,500 ms	Unknown	47	123	Unknown
5	Phillips Achieva OR Siemens TrioTim OR Siemens Prisma_fit	3T	Axial	1.88 mm × 1.88 mm/3.0 mm × 3.0 mm/3.0 mm × 3.0 mm	4.0 mm/4.0 mm/4.0 mm	2,000 ms/2000 ms/2,000 ms	34 ms/30 ms/25 ms	Varied 34–39	144/144/144	Unknown
6	Siemens MAGNETOM Trio	3T	Unknown	4.0 mm × 4.0 mm	4.0 mm	2,500 ms	27 ms	32	varied 130–724	Unknown
7	Siemens Verio	3T	Unknown	3.0 mm × 3.0 mm	3.51 mm	2,500 ms	30 ms	39	198	Unknown

### Data processing

MRI data preprocessing and statistical analyses took place in Analysis of Functional Neuroimages (AFNI v22.1.10) (Cox, 1996) and implemented using afni_proc.py (Example 11b). Anatomical data were skull stripped and normalized to standard Montreal Neurological Institute (MNI) space using non-linear warping with AFNI command @SSwarper and these parameters were applied to the functional data for spatial normalization. The first two volumes of the functional scans were removed, and data were despiked. Volumes were slice time corrected and co-registered to the minimum outlier within the run. Volumes where more than 5% of the brain voxels were considered outliers and were removed from the analysis. In addition, volumes with motion greater than 0.2 mm within a volume were censored and removed from the analysis. Nuisance variables included motion parameters (3 translation, 3 rotation), average ventricle signal, and average white matter signal. Ventricle signals were estimated by combining an MNI ventricle mask with the participant’s cerebral spinal fluid mask derived from the anatomic images. Using multiple regression, a residual time series was calculated for each voxel. The residual time series was then smoothed with a 4 mm FWHM Gaussian kernel, resampled to a 2.5 × 2.5 × 2.5 mm grid, and transformed to MNI space.

### Quality control process

**Figure 1:**
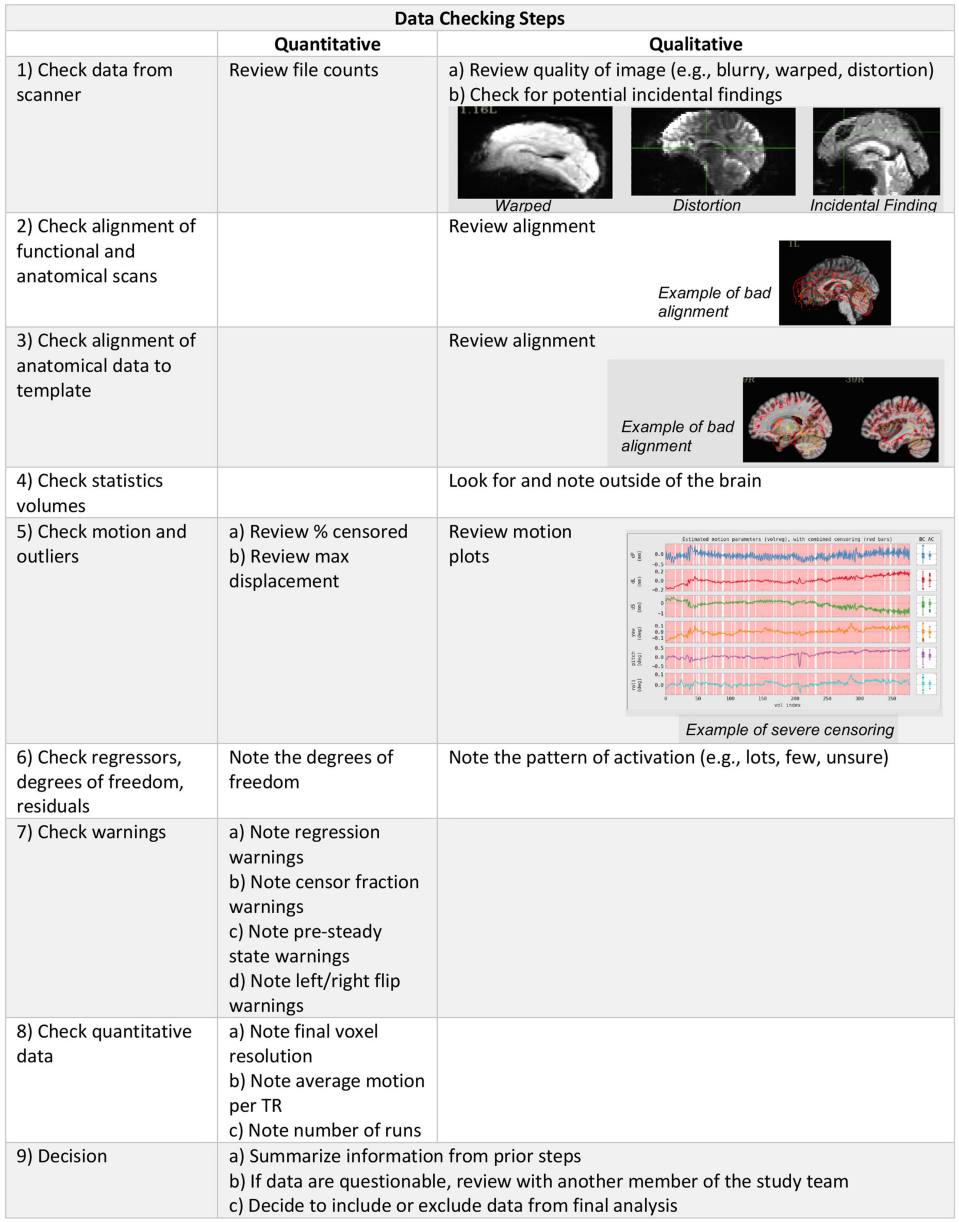
Data checking steps include qualitative and quantitative evaluation of the imaging data to determine inclusion in group level analysis.

Data quality was determined using a combination of quantitative metrics and qualitative assessment (Figure 1). Quantitative metrics included verification of final voxel resolution and outputs from AFNI’s APQC of average motion per TR, max motion displacement, and censor fraction. Quantitative metrics were recorded in our REDCap QC checklist (see supplement) for ease of summary and comparison across participants.

Qualitatively, data were viewed by trained staff who made inclusion/exclusion decisions. Training of staff included walking through each step of our REDCap QC checklist (see supplement) and implementing a double data check system where new staff and trained staff both check and verify the same datasets. Staff were considered trained after inclusion/exclusion decisions were consistent with those made by trained/established staff. This method is a step-by-step approach to reviewing data and documenting the results of each of these steps utilizing a standardized REDCap form. This approach is easy to train new raters – we have successfully trained people across all levels of education, from high school students to those with PhDs – and the double-data entry step facilitates inter-rater reliability assessment. Data entry into REDCap also allows summary data to be easily compiled across participants, and if the checklist is used across multiple studies, data can be easily compared across projects. The inclusion of image examples of poor quality data within our REDCap checklist should improve the replicability and inter-rater reliability as well.

Raw DICOM files were converted to NIFTI format prior to being shared publicly, however, when starting from raw DICOM files, our QC process begins with a verification of data completeness comparing file count, file size, and image acquisition parameters against study protocols. We downloaded the NIFTI files and scrolled through the brain slice by slice within AFNI in order to assess each modality for any acquisition issues, distortion of images, or incidental findings. @SSwarper outputs were visually inspected for good alignment (clear match between the skull-stripped brain and the MNI base template space) and skull-stripping (little to no clipped/missing brain data) prior to being processed with the individual data set afni_proc.py script. We then followed AFNI’s standard processing guidelines to check the processed data using the afni.proc.py quality control output. REDCap QC included checking the APQC and recording of following: excessive motion, warping, and distortion of the original data, alignment issues between the epi to anatomy and anatomy to the MNI template, inspection of the statistics volumes for excessive noise within and outside of the brain, excessive motion, low degrees of freedom, warnings, and a brief summary of the @ss_review_basic. Motion and warnings regarding the severity of the overall censor fraction were recorded at three thresholds based on AFNI warning levels (excluding severe censoring >50%, excluding medium censoring >20%, excluding mild censoring >10%).

In addition to the steps described above, which follow standard processing guidelines from AFNI, if excessive motion was present (>20% censoring), we further checked the epi using @epi_review to visually inspect each run. If major alignment or warping issues were present, we used the @ss_review_driver to visualize the data and troubleshoot challenges in the pre-processing steps. This additional visual inspection process may help identify when a script failed and provide visualization of slices that may not be shown in the APQC file. Data were considered usable if there were no incidental findings, if the functional images were clear with little to no warping or blurring, and if the functional images were well aligned with both the anatomic images and the template. Data were excluded if the preprocessing scripts did not successfully complete after three attempts.

## Results

**Figure 2:**
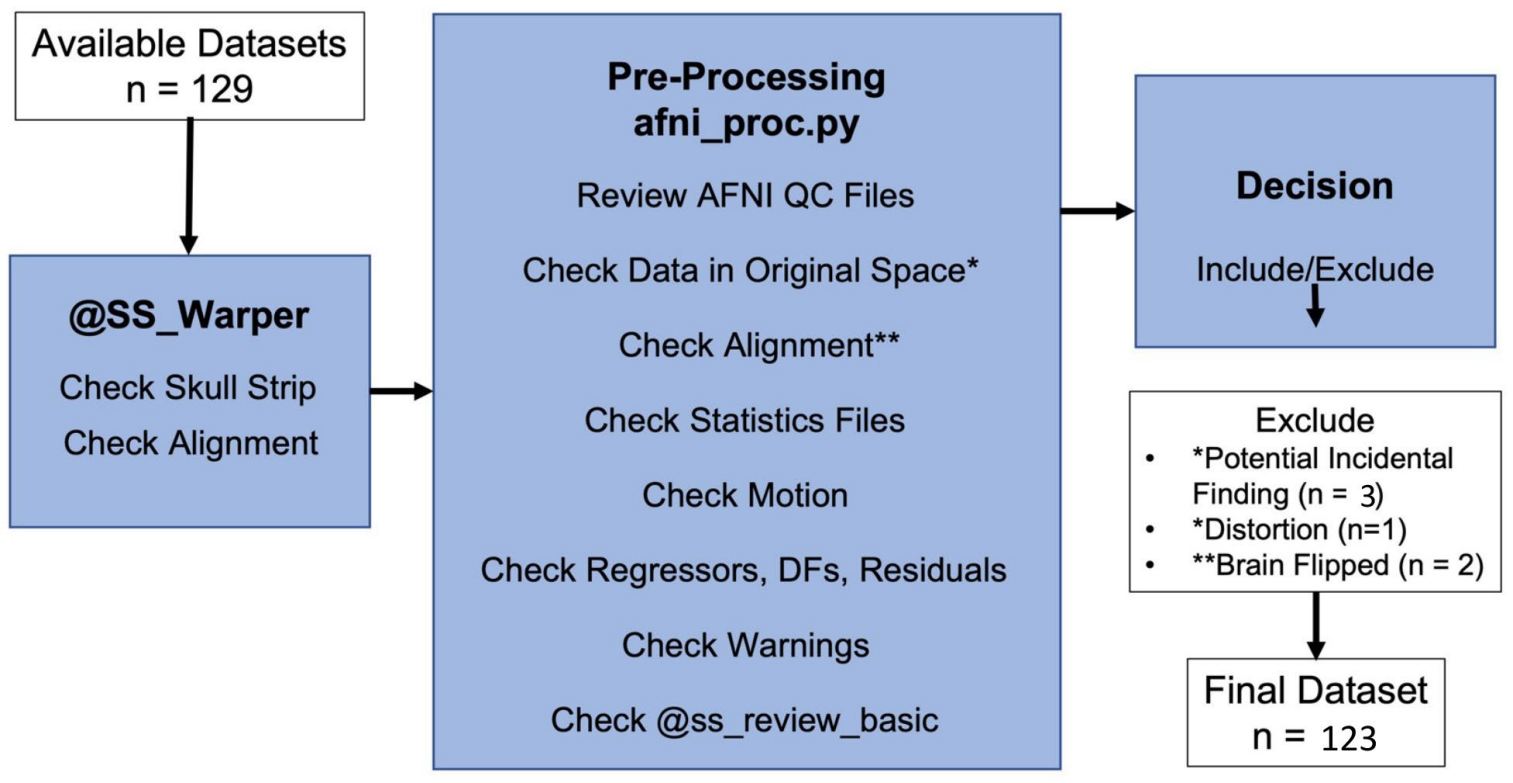
Resting state fMRI data processing and QC workflow.

Of the 129 available data sets, six data sets were excluded due to (A) Script did not complete successfully (*n* = 2), (B) Distortion in the functional image (*n* = 1), or (C) Incidental findings (*n* = 3; Table 2). No data sets were excluded due to motion, leaving 123 data sets to be included for subsequent analysis (Figure 2). The QC data set contained relatively low levels of motion in terms of quantitative metrics: total censor fraction (Mean = 11%, SD = 17%) and max displacement (Mean = 1.25 mm, SD = 0.77 mm). Despite a relatively low censor fraction and max displacement, 30.9% of the data sets had mild censoring or greater (>10%), 14.6% had medium censoring or greater (>20%), and 6.5% of the data sets had severe censoring greater than 50% (Table 3).

**Table 2:** Excluded resting state data sets.

ID	Exclude	QC criteria failed (rationale)	Notes/Examples
315	X	C (incidental finding, black hole in epi file)	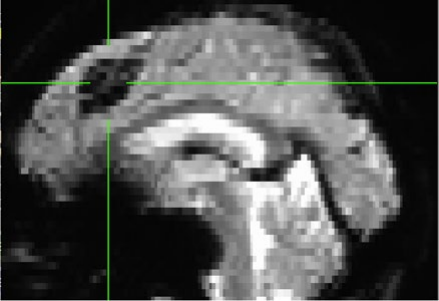
405	X	C (incidental finding, black hole in epi file)	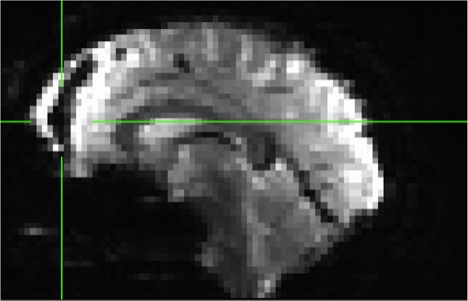
409	X	B (distortion in the epi file)	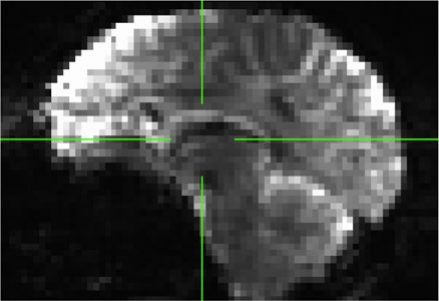
518	X	A (brain was flipped, script failed 3+ times)	During the volume registration step the functional data flipped and problem could not be resolved
519	X	A (brain was flipped, script failed 3+ times)	During the volume registration step the functional data flipped and problem could not be resolved
716	X	C (incidental finding, atrophy and lesions in epi file)	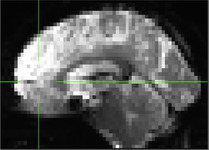

**Table 3:** Resting state data sets exceeding quantitative QC criteria for motion by severity level.

ID	Mild censoring (>10%)	Medium censoring (>20%)	Severe censoring (>50%)
101	X	X	
102	X		
104	X	X	
105	X		
106	X	X	
107	X	X	
109	X		
111	X		
112	X		
114	X		
208	X		
214	X		
307	X	X	X
309	X	X	
314	X	X	
316	X	X	X
402	X		
408	X		
422	X		
502	X		
504	X		
506	X		
507	X	X	X
508	X		
509	X		
511	X	X	
512	X	X	
601	X		
620	X	X	
701	X	X	
703	X	X	X
705	X	X	
706	X	X	X
708	X	X	X
710	X		
712	X	X	X
713	X	X	
714	X	X	X
715	X	X	
Totals	39	21	8

## Discussion

The QC approach described above avoids the use of thresholds for excluding participants and favors inclusion of as many data sets as possible and emphasizes qualitative approaches to QC. A variety of QC approaches can be used to determine data quality and ultimately inclusion or exclusion of a fMRI data set in group analysis, and there are no standards for reporting qualitative approaches. Image artifacts, incidental anatomic findings, and alignment failures that may cause mislocalization of functional data in anatomic space are our primary reasons for excluding data. These features may be missed if only quantitative metrics are used to evaluate data quality. Global metrics such as homogeneity and censoring are unlikely to vary if an image is flipped upside down or if there is an area of localized hypointensity on the BOLD images indicating a potential incidental finding.

Regarding motion, rather than removing entire data sets from group analysis based on excessive censoring as is commonly done, we advocate for relying on within participant censoring and scrubbing methods to clean motion-related artifacts. There is a non-inclusion aspect as well as real dollar cost when excluding data. Often funded by grants, research money is spent recruiting participants, acquiring data, and paying staff to analyze those data. In addition, participants have volunteered their time into studies. Hence, we as researchers have a social and financial obligation to use the data we have collected to the fullest extent and to get the greatest power out of them that we can. This dataset had relatively little motion; however, nearly 15% would have been excluded had we used a threshold approach at medium (>20%) censoring. We have successfully used this inclusive approach in several studies where motion was a greater concern, including studies in a pediatric population (Lepping et al., 2015, 2019).

Some aspects of motion are more challenging to compensate. Minimizing participant motion at data acquisition is ideal; however, this is not realistic in all situations. Several publications offer methods for prospective motion correction for echo-planar imaging (EPI) (Muraskin et al., 2013; Herbst et al., 2015; Maziero et al., 2020). This is achieved by using an in-scanner camera for head tracking to measure head motion in real time and prospectively adjusting the acquisition positioning accordingly. Other useful methods have been developed for fMRI to adjust acquisition positioning during scanning by measuring and correcting for head motion in real time and prospectively for EPI sequences and with further improvement when combined with retrospective motion correction methods (Lee et al., 1998; Thesen et al., 2000; Beall and Lowe, 2014; Lanka and Deshpande, 2019). While not perfect, some of these prospective methods have been successfully used in resting-state functional connectivity analyses (Lanka and Deshpande, 2019), however, these methods are not available for all researchers, and additional sequence and statistical considerations are still needed.

Many of the imaging analysis software packages have added QC tools that have made it easier to assess data quality and report standard quality metrics across packages. AFNI’s APQC html output solidified many of the quality assessment steps we were doing already, including many of the qualitative visual inspection steps. Additional functionality, if provided in the software packages, would further improve the QC process. First, the APQC html file does not currently support saving the data checking within the file itself. Because of this, we have used a separate tool, our REDCap checklist to house the assessments. Second, we have incorporated examples of poor quality data within our REDCap checklist. If that were included in the software output, raters could easily see what the data should not look like, and training for qualitative assessment would be more consistent. Next, other tools within AFNI create QC output files that indicate whether alignment or other downstream steps are likely to fail. Adding that to the APQC process would be useful. Finally, we use the REDCap checklist and project database to export summary QC data for an entire project. It would be helpful to have a group summary QC output directly from the analysis software.

## Conclusion

While quantitative QC metrics including motion are important data to consider when assessing fMRI data quality, some data quality issues may be missed if only quantitative assessments are conducted. Our use of visual inspection throughout the data analysis process ensures that anatomic incidental findings, image artifacts, and processing errors are removed prior to group analysis. Our REDCap checklist can be used to facilitate training of staff and reporting image quality.

## Data availability statement

Publicly available datasets were analyzed in this study. This data can be found at: https://osf.io/qaesm/wiki/home/. Analysis scripts used in this manuscript can be found here: https://github.com/rlepping/kumc-hbic/tree/rsfMRI-qc-paper.

## Ethics statement

All procedures involving human participants were performed in accordance with the ethical standards of the Declaration of Helsinki, and the study was approved by the Institutional Review Board where the data were collected. Informed consent was obtained from all participants.

## Author contributions

RL, H-WY, BM, WB, JH, and LM conceived and designed the approach. RL, H-WY, BM, JH, RK, VP, and LM contributed to the analysis. All authors contributed to the interpretation of the data, drafting and revising of the manuscript, and provide approval for the manuscript.

## Funding

This work was funded in part by the Hoglund Biomedical Imaging Center—which is supported by a generous gift from Forrest and Sally Hoglund—and funding from the National Institutes of Health: S10 RR29577 to the Hoglund Biomedical Imaging Center, UL1 TR000001 to the Frontiers: Heartland Institute for Clinical and Translational Research (CTSA), and P30 AG035982 to the University of Kansas Alzheimer’s Disease Research Center (KU ADRC).

## Conflict of interest

The authors declare that the research was conducted in the absence of any commercial or financial relationships that could be construed as a potential conflict of interest.

## Publisher’s note

All claims expressed in this article are solely those of the authors and do not necessarily represent those of their affiliated organizations, or those of the publisher, the editors and the reviewers. Any product that may be evaluated in this article, or claim that may be made by its manufacturer, is not guaranteed or endorsed by the publisher.

## References

[ref1] Andellini M. Cannatà V. Gazzellini S. Bernardi B. Napolitano A. (2015). Test-retest reliability of graph metrics of resting state MRI functional brain networks: a review. J. Neurosci. Methods 253, 183–192. doi: 10.1016/j.jneumeth.2015.05.020, PMID: 26072249

[ref2] Beall E. B. Lowe M. J. (2014). SimPACE: generating simulated motion corrupted BOLD data with synthetic-navigated acquisition for the development and evaluation of SLOMOCO: a new, highly effective slicewise motion correction. NeuroImage 101, 21–34. doi: 10.1016/j.neuroimage.2014.06.038, PMID: 24969568 PMC4165749

[ref3] Birn R. M. Molloy E. K. Patriat R. Parker T. Meier T. B. Kirk G. R. . (2013). The effect of scan length on the reliability of resting-state fMRI connectivity estimates. NeuroImage 83, 550–558. doi: 10.1016/j.neuroimage.2013.05.099, PMID: 23747458 PMC4104183

[ref4] Biswal B. B. Mennes M. Zuo X. N. Gohel S. Kelly C. Smith S. M. . (2010). Toward discovery science of human brain function. Proc. Natl. Acad. Sci. U. S. A. 107, 4734–4739. doi: 10.1073/pnas.0911855107, PMID: 20176931 PMC2842060

[ref5] Carp J. (2013). Optimizing the order of operations for movement scrubbing: comment on Power et al. NeuroImage 76, 436–438. doi: 10.1016/j.neuroimage.2011.12.061 22227884

[ref6] Chen S. Ross T. J. Chuang K. S. Stein E. A. Yang Y. Zhan W. (2010). A new approach to estimating the signal dimension of concatenated resting-state functional MRI data sets. Magn. Reson. Imaging 28, 1344–1352. doi: 10.1016/j.mri.2010.04.002, PMID: 20655157 PMC2963691

[ref7] Couvy-Duchesne B. Blokland G. A. M. Hickie I. B. Thompson P. M. Martin N. G. de Zubicaray G. I. . (2014). Heritability of head motion during resting state functional MRI in 462 healthy twins. NeuroImage 102, 424–434. doi: 10.1016/j.neuroimage.2014.08.010, PMID: 25132021 PMC4252775

[ref8] Couvy-Duchesne B. Ebejer J. L. Gillespie N. A. Duffy D. L. Hickie I. B. Thompson P. M. . (2016). Head motion and inattention/hyperactivity share common genetic influences: implications for fMRI studies of ADHD. PLoS One 11:e0146271. doi: 10.1371/journal.pone.0146271, PMID: 26745144 PMC4712830

[ref9] Cox R. W. (1996). AFNI: software for analysis and visualization of functional magnetic resonance neuroimages. Comput. Biomed. Res. 29, 162–173. doi: 10.1006/cbmr.1996.0014, PMID: 8812068

[ref10] Di Martino A. Yan C.-G. Li Q. Denio E. Castellanos F. X. Alaerts K. . (2014). The autism brain imaging data exchange: towards a large-scale evaluation of the intrinsic brain architecture in autism. Mol. Psychiatry 19, 659–667. doi: 10.1038/mp.2013.78, PMID: 23774715 PMC4162310

[ref11] Esteban O. Birman D. Schaer M. Koyejo O. O. Poldrack R. A. Gorgolewski K. J. (2017). MRIQC: advancing the automatic prediction of image quality in MRI from unseen sites. PLoS One 12:e0184661. doi: 10.1371/journal.pone.0184661, PMID: 28945803 PMC5612458

[ref12] Fox M. D. Greicius M. (2010). Clinical applications of resting state functional connectivity. Front. Syst. Neurosci. 4:19. doi: 10.3389/fnsys.2010.00019 20592951 PMC2893721

[ref13] Griffanti L. Salimi-Khorshidi G. Beckmann C. F. Auerbach E. J. Douaud G. Sexton C. E. . (2014). ICA-based artefact removal and accelerated fMRI acquisition for improved resting state network imaging. NeuroImage 95, 232–247. doi: 10.1016/j.neuroimage.2014.03.034, PMID: 24657355 PMC4154346

[ref14] Herbst M. Zahneisen B. Knowles B. Zaitsev M. Ernst T. (2015). Prospective motion correction of segmented diffusion weighted EPI. Magn. Reson. Med. 74, 1675–1681. doi: 10.1002/mrm.25547, PMID: 25446934 PMC4451442

[ref15] Johnstone T. Ores Walsh K. S. Greischar L. L. Alexander A. L. Fox A. S. Davidson R. J. . (2006). Motion correction and the use of motion covariates in multiple-subject fMRI analysis. Hum. Brain Mapp. 27, 779–788. doi: 10.1002/hbm.20219 16456818 PMC6871380

[ref16] Kong X. Z. Zhen Z. Li X. Lu H. H. Wang R. Liu L. . (2014). Individual differences in impulsivity predict head motion during magnetic resonance imaging. PLoS One 9:e104989. doi: 10.1371/journal.pone.0104989, PMID: 25148416 PMC4141798

[ref17] Lanka P. Deshpande G. (2019). Combining prospective acquisition CorrEction (PACE) with retrospective correction to reduce motion artifacts in resting state fMRI data. Brain Behav. 9:e01341. doi: 10.1002/brb3.1341, PMID: 31297966 PMC6710196

[ref18] Lazar N. A. (2008). “The statistical analysis of functional MRI data” in Statistics for biology and health. ed. Gail M. (New York, NY: Springer), 299.

[ref19] Lee C. C. Grimm R. C. Manduca A. Felmlee J. P. Ehman R. L. Riederer S. J. . (1998). A prospective approach to correct for inter-image head rotation in fMRI. Magn. Reson. Med. 39, 234–243. doi: 10.1002/mrm.1910390210, PMID: 9469706

[ref20] Lepping R. J. Bruce A. S. Francisco A. Yeh H. W. Martin L. E. Powell J. N. . (2015). Resting-state brain connectivity after surgical and behavioral weight loss. Obesity (Silver Spring) 23, 1422–1428. doi: 10.1002/oby.21119, PMID: 26053145 PMC4483156

[ref21] Lepping R. J. Honea R. A. Martin L. E. Liao K. Choi I. Y. Lee P. . (2019). Long-chain polyunsaturated fatty acid supplementation in the first year of life affects brain function, structure, and metabolism at age nine years. Dev. Psychobiol. 61, 5–16. doi: 10.1002/dev.21780, PMID: 30311214 PMC6317998

[ref22] Little R. J. A. Rubin D. B. (eds). (2002). “Statistical analysis with missing data” in Wiley Series in probability and statistics. 2nd ed (New York, NY: Wiley), 381.

[ref23] Markiewicz C. J. Gorgolewski K. J. Feingold F. Blair R. Halchenko Y. O. Miller E. . (2021). The OpenNeuro resource for sharing of neuroscience data. Elife 10:10. doi: 10.7554/eLife.71774 PMC8550750 34658334

[ref24] Maziero D. Rondinoni C. Marins T. Stenger V. A. Ernst T. (2020). Prospective motion correction of fMRI: improving the quality of resting state data affected by large head motion. NeuroImage 212:116594. doi: 10.1016/j.neuroimage.2020.116594, PMID: 32044436 PMC7238750

[ref25] Muraskin J. Ooi M. B. Goldman R. I. Krueger S. Thomas W. J. Sajda P. . (2013). Prospective active marker motion correction improves statistical power in BOLD fMRI. NeuroImage 68, 154–161. doi: 10.1016/j.neuroimage.2012.11.052, PMID: 23220430 PMC3638742

[ref26] Pardoe H. R. Kucharsky Hiess R. Kuzniecky R. (2016). Motion and morphometry in clinical and nonclinical populations. NeuroImage 135, 177–185. doi: 10.1016/j.neuroimage.2016.05.005, PMID: 27153982

[ref27] Patriat R. Molloy E. K. Birn R. M. (2015). Using edge voxel information to improve motion regression for rs-fMRI connectivity studies. Brain Connect 5, 582–595. doi: 10.1089/brain.2014.0321, PMID: 26107049 PMC4652211

[ref28] Patriat R. Reynolds R. C. Birn R. M. (2016). An improved model of motion-related signal changes in fMRI. NeuroImage 144, 74–82. doi: 10.1016/j.neuroimage.2016.08.051 27570108 PMC5533292

[ref29] Power J. D. Barnes K. A. Snyder A. Z. Schlaggar B. L. Petersen S. E. (2012). Spurious but systematic correlations in functional connectivity MRI networks arise from subject motion. NeuroImage 59, 2142–2154. doi: 10.1016/j.neuroimage.2011.10.018, PMID: 22019881 PMC3254728

[ref30] Power J. D. Barnes K. A. Snyder A. Z. Schlaggar B. L. Petersen S. E. (2013). Steps toward optimizing motion artifact removal in functional connectivity MRI; a reply to Carp. NeuroImage 76, 439–441. doi: 10.1016/j.neuroimage.2012.03.017, PMID: 22440651 PMC3834590

[ref31] Power J. D. Mitra A. Laumann T. O. Snyder A. Z. Schlaggar B. L. Petersen S. E. (2014). Methods to detect, characterize, and remove motion artifact in resting state fMRI. NeuroImage 84, 320–341. doi: 10.1016/j.neuroimage.2013.08.048, PMID: 23994314 PMC3849338

[ref32] Power J. D. Schlaggar B. L. Petersen S. E. (2015). Recent progress and outstanding issues in motion correction in resting state fMRI. NeuroImage 105, 536–551. doi: 10.1016/j.neuroimage.2014.10.044, PMID: 25462692 PMC4262543

[ref33] Pruim R. H. Mennes M. Buitelaar J. K. Beckmann C. F. (2015). Evaluation of ICA-AROMA and alternative strategies for motion artifact removal in resting state fMRI. NeuroImage 112, 278–287. doi: 10.1016/j.neuroimage.2015.02.063, PMID: 25770990

[ref34] Rogers B. P. Morgan V. L. Newton A. T. Gore J. C. (2007). Assessing functional connectivity in the human brain by fMRI. Magn. Reson. Imaging 25, 1347–1357. doi: 10.1016/j.mri.2007.03.007 17499467 PMC2169499

[ref35] Satterthwaite T. D. Elliott M. A. Gerraty R. T. Ruparel K. Loughead J. Calkins M. E. . (2013). An improved framework for confound regression and filtering for control of motion artifact in the preprocessing of resting-state functional connectivity data. NeuroImage 64, 240–256. doi: 10.1016/j.neuroimage.2012.08.052, PMID: 22926292 PMC3811142

[ref36] Siegel J. S. Power J. D. Dubis J. W. Vogel A. C. Church J. A. Schlaggar B. L. . (2014). Statistical improvements in functional magnetic resonance imaging analyses produced by censoring high-motion data points. Hum. Brain Mapp. 35, 1981–1996. doi: 10.1002/hbm.22307 23861343 PMC3895106

[ref37] Thesen S. Heid O. Mueller E. Schad L. R. (2000). Prospective acquisition correction for head motion with image-based tracking for real-time fMRI. Magn. Reson. Med. 44, 457–465. doi: 10.1002/1522-2594(200009)44:3<457::AID-MRM17>3.0.CO;2-R, PMID: 10975899

[ref38] Thulborn K. R. (1999). Visual feedback to stabilize head position for fMRI. Magn. Reson. Med. 41, 1039–1043. doi: 10.1002/(SICI)1522-2594(199905)41:5<1039::AID-MRM24>3.0.CO;2-N, PMID: 10332888

[ref39] Van Dijk K. R. Sabuncu M. R. Buckner R. L. (2012). The influence of head motion on intrinsic functional connectivity MRI. NeuroImage 59, 431–438. doi: 10.1016/j.neuroimage.2011.07.044, PMID: 21810475 PMC3683830

[ref40] Vanderwal T. Kelly C. Eilbott J. Mayes L. C. Castellanos F. X. (2015). Inscapes: a movie paradigm to improve compliance in functional magnetic resonance imaging. NeuroImage 122, 222–232. doi: 10.1016/j.neuroimage.2015.07.069, PMID: 26241683 PMC4618190

